# Young’s Modulus of Different Illitic Clays during Heating and Cooling Stage of Firing

**DOI:** 10.3390/ma13214968

**Published:** 2020-11-04

**Authors:** Tomáš Húlan, Igor Štubňa, Ján Ondruška, Štefan Csáki, František Lukáč, Marek Mánik, Libor Vozár, Jurijs Ozolins, Tiit Kaljuvee, Anton Trník

**Affiliations:** 1 Department of Physics, Faculty of Natural Sciences, Constantine the Philosopher University in Nitra, Tr. A. Hlinku 1, 94974 Nitra, Slovakia; thulan@ukf.sk (T.H.); istubna@ukf.sk (I.Š.); jondruska@ukf.sk (J.O.); marek.manik@ukf.sk (M.M.); lvozar@ukf.sk (L.V.); 2 Institute of Plasma Physics, Czech Academy of Sciences, Za Slovankou 3, 18200 Prague, Czech Republic; csaki@ipp.cas.cz (Š.C.); lukac@ipp.cas.cz (F.L.); 3 Building Testing and Research Institute, Braneckého 2, 94901 Nitra, Slovakia; 4 Rudolfs Cimdins Riga Biomaterials Innovations and Development Centre of RTU, Institute of General Chemical Engineering, Faculty of Materials Science and Applied Chemistry, Riga Technical University, Pulka 3, LV-1007 Riga, Latvia; jurijs.ozolins@rtu.lv; 5 Laboratory of Inorganic Materials, School of Engineering, Tallinn University of Technology, Ehitajate tee 5, 19086 Tallinn, Estonia; Tiit.Kaljuvee@ttu.ee; 6 Department of Materials Engineering and Chemistry, Faculty of Civil Engineering, Czech Technical University in Prague, Thákurova 7, 16629 Prague, Czech Republic

**Keywords:** clay, illite, quartz, thermal expansion, Young’s modulus

## Abstract

Dynamical thermomechanical analysis of 5 illite-based clays from deposits in Slovakia, Estonia, Latvia, and Hungary is presented. The clays consist of illite (37–80 mass%), quartz (12–48 mass%), K-feldspar (4–13 mass%), kaolinite (0–18 mass%), and calcite (0–3 mass%). Young’s modulus is measured during the heating and cooling stages of firing (25 °C → 1100 °C → 25 °C). The liberation of the physically bound water increases Young’s modulus by ∼70% for all studied clays. By increasing the temperature, dehydroxylation and the α → β transition of quartz take place without a significant effect on Young’s modulus. Sintering, which starts at 800 °C, leads to an intensive increase in Young’s modulus up to the highest temperature (1100 °C). The increase remains also in the early stage of cooling (1100 °C → 800 °C). This increase of Young’s modulus is also the result of solidification of the glassy phase, which is finished at ∼750 °C. A sharp minimum of Young’s modulus is observed at around the β → α transition of quartz. Then, Young’s modulus still decreases its value down to the room temperature. The physical processes observed during heating and cooling do not differ in nature for the studied clays. Values of Young’s modulus vary at around 8 GPa, up to 800 °C. During sintering, Young’s modulus reaches values from 30 GPa to 70 GPa for the studied clays. The microstructure and composition given by the origin of the clay play a cardinal role for the Young’s modulus of the final ceramic body.

## 1. Introduction

Traditional ceramics made from natural mineral sources (clays, quartz, and feldspars) are still used in many fields. A plastic mixture of these materials with water is the base of the most used forming method of ceramic bodies, which are sequentially dried and fired. Some innovative materials based on illitic clays were investigated in the past decade: ceramic hollow spheres for agriculture and civil engineering [1,2] or lightweight cenosphere–clay syntactic-foam [3]. Illitic clay was the preferred choice as a high-temperature inorganic binder in hybrid materials [4]. Quartz, which is always present in traditional ceramics, plays an essential role during firing and influences the fired bodies’ mechanical properties to a great extent [5,6].

There are large areas in Europe where kaolin is rare or completely missing. Here, the ceramic industry is based on illitic clays. That is why the investigation of the firing of illitic clays is important and can provide advantageous information. This paper intends to provide a comprehensive overview of the development of Young’s modulus, thermal expansion, mass change, and bulk density during the firing of illitic clays originating in Slovakia, Hungary, Estonia, and Latvia [7,8,9,10,11,12,13]. The result will contribute to understanding of the heat-induced processes through the in situ measurement of Young’s modulus.

Young’s modulus (*E*) is a mechanical quantity of great importance for ceramics. It depends on external influences, e.g., temperature, as well as on the intrinsic properties of the studied material. Therefore, Young’s modulus allows an indirect study of the microstructure (porosity or texture). Influences of the preparation technology (mineral composition, forming, drying, firing) on ceramic materials and Young’s modulus are suitable for the experimental study of the crack formation in ceramic bodies [6,9]. Notable progress for studying the formation of the cracks brought the combination of the acoustic emission and Young’s modulus, both measured during firing [6,8,9].

According to Griffith’s theory, Young’s modulus is directly proportional to the mechanical strength [14]. The proportionality between them also follows directly from Hooke’s law [15]. Consequently, the obtained results can help give a picture of the mechanical strength.

### 1.1. Quartz and Illite

Quartz is the most abundant mineral in Earth’s crust due to its high stability, which is determined by the bound energy of Si–O (368 kJ/mol) [16]. Around the temperature of 573 °C, quartz undergoes a reversible structural modification which is connected with ∼0.7% volume expansion when heated or contraction when cooled [5,17]. While the low-temperature α-quartz expands up to 573 °C, the high-temperature β-quartz slightly contracts above this temperature. The densities of quartz are *ρ_α_* (20 °C) = 2.65 g/cm^3^ and *ρ_β_* (590 °C) = 2.53 g/cm^3^ [17]. Other physical properties of quartz also show anomalous behavior around its α ↔ β transition. For example, Young’s modulus and Poisson’s ratio pass through a sharp V-shape minimum in their temperature dependences [18,19]. A steep decrease in the value of specific heat capacity was also obtained [17]. The α ↔ β transition of quartz takes place in a narrow temperature interval around 573 °C, and the enthalpy of this transition is ∼45.4 kJ/mol [20].

Illite is a significant rock-forming mineral and is the main component of illitic clay. Its structure consists of a repetition of tetrahedron–octahedron–tetrahedron (T-O-T) sheets in one layer. The interlayer space is occupied mainly by potassium cations, which are responsible for the absence of swelling. Additionally, a variable amount of water molecules lies between the T-O-T layers [21,22,23,24]. Generally, during heating up to 1200 °C, illite goes through several changes. The first is the loss of physically bound water between the room temperature and 300 °C [25]. The next process is dehydroxylation, which begins at ∼450 °C [25,26,27,28], is accompanied by a mass loss (5.2–5.6) mass% [25], and no shrinkage [27,29]. Concerning the reaction mechanism of the illite dehydroxylation, it is still not possible to reach a uniform definition [26]. Differential thermal analysis (DTA) and thermogravimetric analysis (TG) confirm a two-step dehydroxylation with two values of activation energy: 676 kJ/mol for the first step and 231 kJ/mol for the second step [26]. Illite reflections in the XRD pattern survive during heating up to ∼950 °C when starting their disappearance at ∼1050 °C [27,30,31]. The high-temperature reactions are accompanied by a steep contraction [29,30] and a formation of new phases, e.g., Al-Si spinel, mullite, and amorphous phase [32].

Pure illite does not occur in nature. For example, illitic clay from the Tokaj region in Hungary, which is composed of illite (80 mass%), montmorillonite (4 mass%), quartz (12 mass%), and orthoclase (4 mass%), is considered as a good reference material for studying the properties of illite [22,24,26,30,33].

### 1.2. Illite-Based Ceramics

Clay mineral illite is the main component of the mixtures for traditional ceramics (mainly building ceramics and pottery) made from natural illitic clays. Besides illite, quartz, and feldspar are always present in natural illitic clays. The other components often found in illitic clays can be carbonates and iron oxides. Illitic clay can be the base of porous ceramics and can also be used with fly ash [34,35]. Preparation of illite-based ceramics is based on firing the ceramic bodies at temperatures from 700 °C to 1100 °C [36]. After such thermal treatment, the ceramic body becomes rigid, and its mechanical strength reaches sufficient values. An improvement in other essential properties is also observable. The mineral phases found in fired clay are quartz, orthoclase, and newly created phases (glass over 50 mass%), a relatively small amount of the Al-Si spinel, and mullite [32]. The glassy phase’s amount depends on the original mineral composition and the firing temperature. If the maximum firing temperature ranges from 1000 °C to 1100 °C and the original content of illite is below 50 mass%, the glassy phase varies between 50 mass% and 60 mass% [9,11,12,13]. The part of the glassy phase can also reach 80 mass% when the clay with the illite content of more than 80 mass% is used [10,30]. The reason for the high amount of glassy phase in the illite-based ceramics is potassium contained in the illite structure, which is a very effective flux [37].

This paper aims to show differences between thermogravimetric, thermodilatometric, and elastic behavior of illitic clays from different regions. The previous studies [7,10,11,12,13]) are extended by SEM pictures and the analysis of the cooling stage of firing in this study.

## 2. Materials and Methods 

Natural illitic clays from deposits in Füzérradvány (Tokai region, Hungary), Radobica (Upper Nitra region, Slovakia), Kunda (Viru county, Estonia), Arumetsa (Parnu county, Estonia), and Liepa (Liepa, Latvia) were investigated in previous papers [7,10,11,12,13] which create a basis for this comparative study. Clay samples for the dynamic thermomechanical analysis (D-TMA) were prepared in the same way as a green ceramic body. Lumps of clays were crushed and milled, and the obtained powders were mixed with water to create a plastic mass with ∼30 mass% of the water. All samples were prepared using extrusion of the plastic mass with a rectangular cross-section, except for samples from Radobica clay, where cylindrical samples were extruded; note that the shape of the cross-section does not influence the results. After open air drying, the samples contained equilibrium moisture between 1.5–2.5 mass%. The mineralogical composition of the green clays is given in Table 1 and their chemical composition in Table 2.

Young’s modulus can be successfully determined during heating/cooling with methods based on the measurement of the resonant frequency of the samples with a uniform cross-section. If the fundamental mode of flexural vibration is used, the formula for calculation of Young’s modulus is given in [38,39,40] and has the form

(1) $$E\left(t\right)=K\frac{m_{0}l_{0}^{3}}{d_{0}^{4}}\frac{\left[1+\frac{\Delta m\left(t\right)}{m_{0}}\right]}{\left[1+\frac{\Delta l\left(t\right)}{l_{0}}\right]}f^{2}\left(t\right)\;T,$$

where *K* = 1.12336 for the circular cross-section, *K* = 0.97286 for the square cross-section, and *T* is the correction factor, which is used if *l*_0_/*d*_0_ < 20. Quantities *m*_0_, *l*_0_ are the initial mass and the length of the sample, *d*_0_ is the initial diameter of the circular cross-section or side of the square cross-section, and Δ*l*(*t*)/*l*_0_ and Δ*m*(*t*)/*m*_0_, are the relative linear thermal expansion and relative mass change of the sample measured by thermodilatometer and TG analyzer. The resonant frequency *f* is continuously measured during heating/cooling with the sonic resonant method [41] or the impulse excitation technique [42] using the apparatus made in the Thermophysical laboratory, CPU Nitra [41,42]. Cylindrical samples with dimensions ∅10 × 130 mm^2^ and prismatic samples with dimensions 10 × 10 × 130 mm^3^ were used for D-TMA, which was performed with a heating/cooling rate of 5 °C/min in the firing cycle 20 °C → 1100 °C → 20 °C. The maximum relative expanded uncertainty of the Young’s modulus is ∼2% [42].

To explain the development of Young’s modulus during heating and cooling, additional analyses are useful, particularly thermogravimetry (TG) and thermodilatometry (TDA). TG was carried out using the upgraded DTA/TG analyzer Derivatograph 1100° (MOM Budapest, Hungary) [43] on compact samples with the initial mass of ∼2.5 g. TDA was determined using a horizontal alumina push-rod dilatometer, which was made in the Thermophysical laboratory, CPU Nitra [44]. TG and TDA should be performed in the same temperature regime as D-TMA, using samples with the shape and dimensions close to the D-TMA samples. The heating/cooling rate of 5 °C/min was also used in TDA and TG analyses. Compact samples with dimensions ∅10 × 20 mm^2^ and 10 × 10 × 20 mm^3^ were used for TG. Samples for TDA had dimensions ∅10 × 30 mm^2^ and 10 × 10 × 30 mm^3^. All measurements were done in a static air atmosphere. The high similarity of the sample dimensions and identical thermal regimes permit a comparison of the results obtained on different clays.

The bulk density was calculated from the TG and TDA results to obtain its actual values during heating and cooling according to the formula

(2) $$\rho \left(t\right)=\rho_{0}\frac{\left[1+\frac{\Delta m\left(t\right)}{m_{0}}\right]}{\left[1+3\frac{\Delta l\left(t\right)}{l_{0}}\right]},$$

where *ρ*_0_ is the bulk density of the green sample at room temperature.

Microstructure observations were performed on a FEI QuantaTM FX200 (Thermo Fisher Scientific, Waltham, MA, USA) scanning electron microscope in low vacuum mode (100 Pa) with an accelerating voltage of 10 kV. 

## 3. Results and Discussion

### 3.1. Mass Changes 

Illitic clays undergo several structural and microstructural changes when they were heated from the room temperature up to 1100 °C. These changes were reflected in TG, TDA, and D-TMA results.

**Figure 1:**
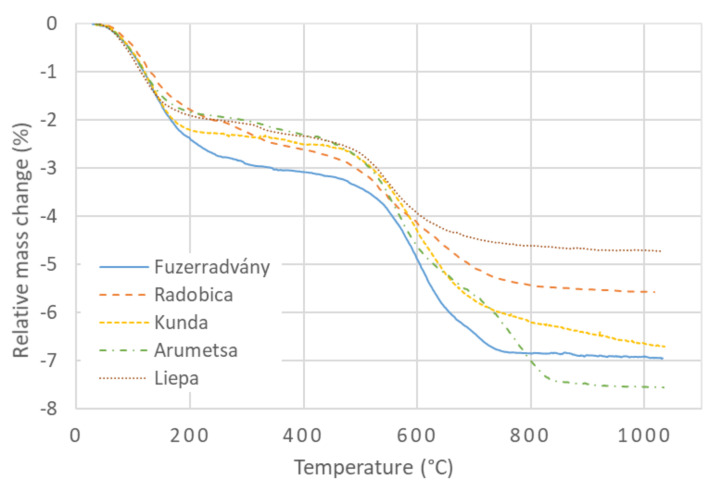
Relative mass change of investigated clays.

The TG curves of the investigated clays are shown in Figure 1. The first significant process was the release of the physically bound water (20 °C–300 °C) from the microstructure. In addition, a small amount of water, which was bound between the T-O-T layers, was released at ∼300 °C [23]. The next process was dehydroxylation of the clay components (illite, kaolinite, montmorillonite, and chlorite), which started at ∼450 °C. Radobica and Arumetsa clays contain calcite; therefore, CO_2_ is released from them due to the calcite decomposition above 700 °C. This is more significant for Arumetsa clay, which contains 3 mass% of calcite. Kunda clay also contains a small amount of pyrite, which decomposes in two steps, the second of them at temperatures above 740 °C [45]. The escape of SO_2_, which was confirmed by evolved gas analysis (EGA) [11], causes a mass loss of the Kunda clay above 750 °C.

Considering the samples were prepared by extruding, some small differences in their microstructure can be expected and their porosity can be different. The samples also contained different parts of clay crystals, which well bind the water on their surfaces. Therefore, the samples’ ability to bind the water was slightly different; i.e., the studied samples contained different amounts of the physically bound water. This is reflected in Figure 1 as a scatter of the TG curves in the low-temperature region. The values of the mass losses at 300 °C revealed that the Füzérradvány clay contained the highest amount of physically bound water. The explanation lies in the presence of the electrical charge on the illite crystal surface, which attracts water molecules. Thus, higher illite content leads to a higher amount of physically bound water in the clays.

When the water from the pores and crystal surfaces was evaporated, the next mass loss was caused by dehydroxylation, which runs in the temperature interval from 450 °C to 700 °C in illite, kaolinite, montmorillonite, and chlorite. This mass loss does not depend on the sample preparation, but only on the part of these minerals in the samples.

### 3.2. Volume Changes

**Figure 2:**
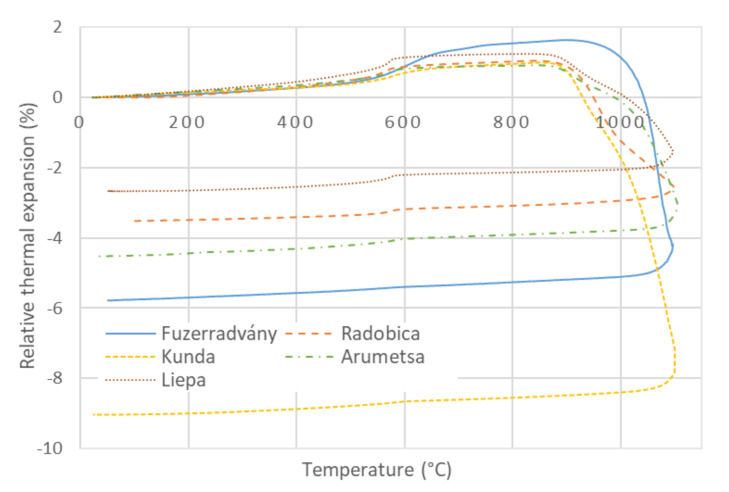
Relative thermal expansion of investigated clays.

The TDA curves of the clays are present in Figure 2. A small expansion was observable at the lowest temperatures (up to 300 °C) in which two processes took place: a) standard thermal expansion, and b) release of the remaining physically bound water. Due to the latter, crystals were setting closer to each other, which causes a contraction. The total volume (dimension) change is a superposition of these two physical processes. When the remaining water release was over, the expected thermal expansion continued until the beginning of dehydroxylation. This process led to an expansion of the minerals with T-O-T structure (illite and montmorillonite) [28]. On the other hand, kaolin (with T-O structure) contracts during dehydroxylation [28]. All clays also contained quartz, which underwent the α → β transition at 573 °C (i.e., in the dehydroxylation background) and were accompanied by an expansion. Therefore, during dehydroxylation, the TDA results were the superposition of expansion (quartz, feldspar, illite, and montmorillonite) and contraction (kaolinite). After dehydroxylation, the common thermal expansion continued up to the start of vitrification and sintering processes. Since densification induced by sintering is much more intensive than the thermal expansion, the overall contraction was registered.

Differences between the TDA curves during heating 20 °C → 800 °C were small, except for the Füzérradvány sample with 80% of illite (see Figure 2). On the other hand, the contraction of the different samples during sintering was significant. The relative contractions were scattered in the range from −2.5% for the Liepa clay to −9% for the Kunda clay.

### 3.3. Bulk Density

**Figure 3:**
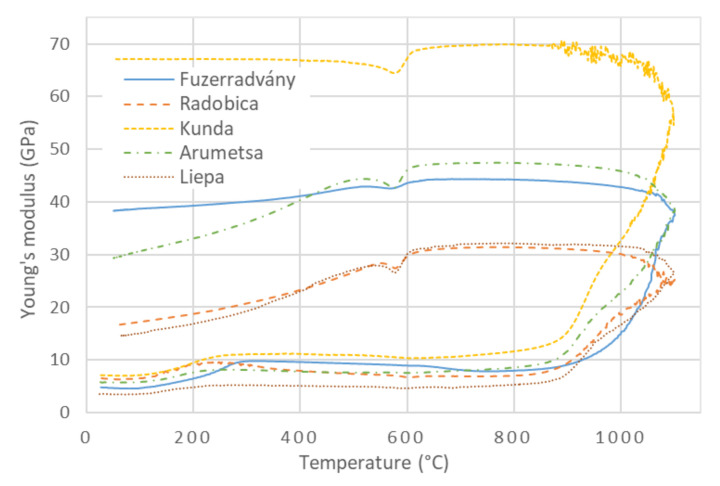
Normalized bulk density of investigated clays.

Since Young’s modulus is directly proportional to the bulk density, it is useful to know the development of this quantity during firing. The bulk density, Figure 3, was calculated from TG and TDA results according to Equation (2). The bulk density’s starting values, which vary from 1.6 g/cm^3^ to 1.9 g/cm^3^, depended on the sample preparation. The composition did not play a significant role because of the densities of crystals (illite 2.7 g/cm^3^, kaolinite 2.6 g/cm^3^, montmorillonite 2.5 g/cm^3^, chlorite 2.8 g/cm^3^, quartz 2.6 g/cm^3^, and feldspar 2.6 g/cm^3^) differ only slightly. The initial values of bulk density were: 1.66 g/cm^3^ for Füzérradvány, 1.85 g/cm^3^ for Radobica, Kunda, and Liepa, and 1.90 g/cm^3^ for Arumetsa.

The bulk density depends on the mass losses up to 700 °C during heating. After the release of all water (both physically bound and constituent), the bulk density depended only on the volume, which changes mainly due to sintering (see Figure 3). The normalized bulk density was calculated as *ρ_f_*/*ρ_g_*, where *ρ_f_* is the bulk density of the fired sample and *ρ_g_* of the green (unfired) sample.

### 3.4. Young’s Modulus during Heating

**Figure 4:**
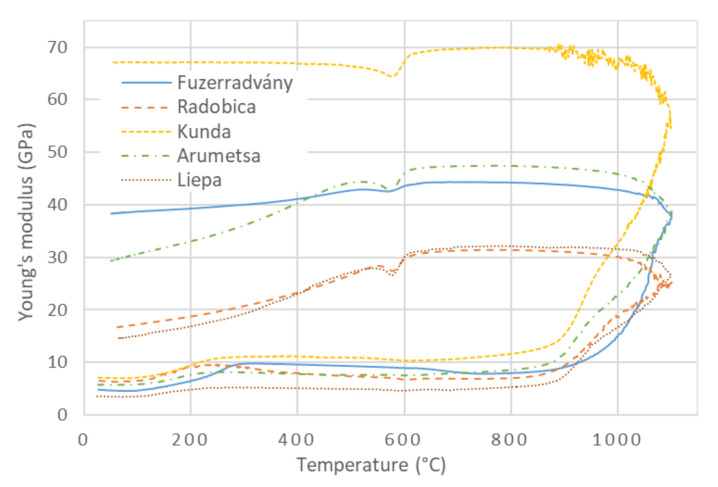
Young’s modulus of investigated clays.

The relationship between Young’s modulus, calculated according to Equation (1), and the temperature for the investigated clays is shown in Figure 4. The Young’s modulus followed the bulk density at the lowest temperatures (20 °C–300 °C) when the release of the physically bound water set the crystals closer to each other, and the mechanical contacts between the crystals were improved. This led to a significant increase in Young’s modulus. An intensive mass loss was completed at 200 °C (Figure 1), but the maximum of Young’s modulus was shifted towards higher temperatures (250 °C–300 °C). It could be caused by the release of the water from the finest pores, in which water molecules were bound tighter, and a higher temperature is needed to release them. Then, Young’s modulus monotonously decreased, showing that neither phase changed nor physical events occurred in the samples between 300 °C and 450 °C. The cause of Young’s modulus’s decrease, in this case, was only a weakening of the interatomic bonds in crystals due to the increased temperature [46].

Dehydroxylation influenced Young’s modulus only to a small extent. The mass loss and thermal expansion resulted in a decrease in the bulk density (Figure 3), which was directly proportional to Young’s modulus. Kaolinite (T-O structure) lost ∼14 mass% of the constituent water and transformed into metakaolinite with a highly defective structure with vacancies [47]. Illite (T-O-T structure) lost (5–6) mass% of the constituent water [25], which also created vacancies in the structure. The results of TDA showed a significant increase in dimensions between 450 °C and 650 °C, which reflected the newly generated defects in the illite structure and the α → β transition of quartz. Dehydroxylation is expected to decrease Young’s modulus of clays. Despite this expectation, no significant decrease in Young’s modulus was observed in the dehydroxylation region. The same could be said about mechanical strength, which is directly proportional to Young’s modulus [15]. The influence of internal defects, created during dehydroxylation inside the illite and kaolinite crystals, on Young’s modulus is compensated by strengthening of the contacts between crystals. Such strengthening can only be caused by solid-state sintering because temperatures from 450 °C to 700 °C were not sufficient for intensive sintering in the presence of the liquid phase.

From the microstructural point of view, the most significant change that occurs in the ceramic body during heating is a decrease of porosity. This partially occurs during the solid-state sintering at temperatures below ∼850 °C and mainly during the liquid-state sintering at temperatures above 900 °C [16,48]. Illitic clays are known for good vitrification [32,49,50], and the glassy phase part in the fired body usually reaches more than 50 mass% (Table 3). The firing conditions were not sufficient to achieve the melting of quartz grains; consequently, quartz grains surrounded with glassy phase remained present in the ceramic microstructure.

Young’s modulus’s initial values were 4 GPa–7 GPa, and the samples’ preparation probably caused these differences. The relationships between Young’s modulus and the temperature had the same main features—the increase in Young’s modulus due to the release of the physically bound water, then a moderate decrease in Young’s modulus without a visible influence of dehydroxylation. Solid-state sintering was evident through the slight increase in Young’s modulus above 700 °C. Finally, a steep increase in Young’s modulus during sintering above 850 °C was registered. Values of Young’s modulus at the highest temperature (1100 °C) differ from 25 GPa (Radobica) to 58 GPa (Kunda).

### 3.5. Young’s Modulus during Cooling

As mentioned above, the clay samples were fired up to 1100 °C. Their new mineral composition can be seen in Table 3. This composition is supposed to be the same during cooling with one exception: β-quartz turns into α-quartz at ∼573 °C.

The mass of the sample remained constant during cooling from 1100 °C. A regular contraction was observed in the cooling stage of the firing; only a step contraction caused by the β → α transition of quartz was visible in the TDA curves (Figure 2). The sample dimensions slightly decreased due to the thermal contraction, and the bulk density slightly increased (Figure 3).

When cooling started, the temperature was high enough for sintering to continue, but the viscosity of the glassy phase had already started to increase. Sintering and increasing viscosity increased Young’s modulus down to the glass transformation temperature (∼750 °C), below which Young’s modulus began to decrease. When the ceramic body became solid, the differences between the linear thermal expansion coefficients (LTEC) of the mineral phases generated local mechanical stress, which is the source of cracking [6].

**Figure 5:**
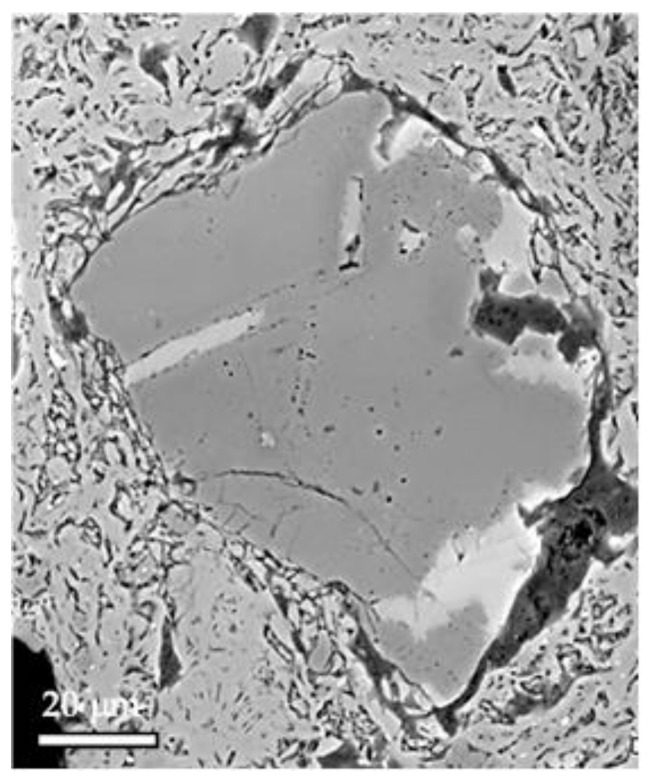
Fracture surface with quartz grain and circumferential crack around it. Abraded, not etched sample (adopted from [9]).

Cracking influences Young’s modulus and the mechanical strength of ceramics to a large extent [51]. The source of cracking was related to the quartz grains, which LTEC significantly changes at the β → α transition, while LTEC of other phases changes very slowly. The circumferential cracking around the quartz grains is well visible via electron microscopy (Figure 5). It is sometimes hypothesized that these cracks are created when the grains shrink rapidly during the β → α transition [52,53]. At first glance, this explanation is quite reasonable and straightforward.

**Figure 6:**
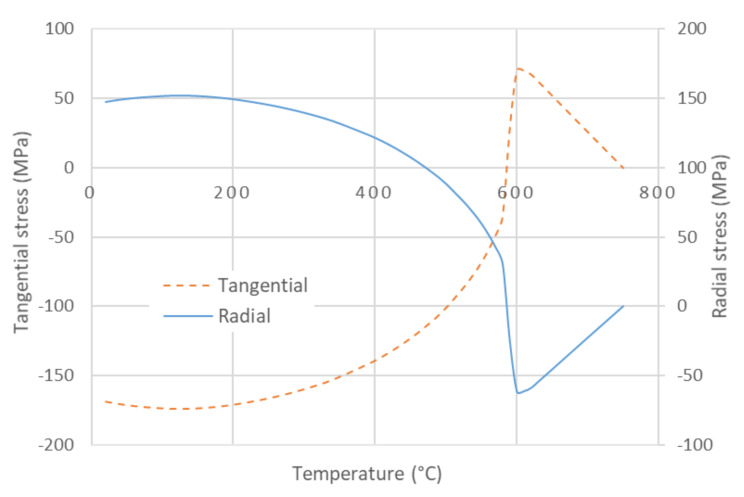
Development of the radial stress and the tangential stress on the quartz grain surface during cooling for 30% part of quartz in the double sphere model. The tensile stress has positive value, the compressive stress is negative.

Young’s modulus, which is sensitive to the creation of cracks, showed that cracking began at the glass transformation temperature (∼750 °C) and continued below 573 °C. A simple model of two concentric spheres, in which the inner sphere is quartz grain and the outer sphere is a glassy phase, predicts that the surface and a close vicinity of the grain are exposed to tensile tangential stress and compressive radial stress at temperatures above 573 °C (Figure 6) [54]. When the cooling continued, a quick alteration of the stresses took place in the narrow interval at around 573 °C. The tangential stress becomes compressive and the radial stress becomes tensile for temperatures less than 560 °C (Figure 6). The tensile stresses were sources of the cracks. Such stresses are always present except for the short interval when the stresses alter. No stress acts on the grain in this interval, and no new cracks were formed [6,8]. This was confirmed by a temporary recovery of Young’s modulus; see the V-shaped minima in Figure 4. A very similar course of Young’s modulus of ceramic clay in the heating and cooling stage of the firing as those given in Figure 4 was obtained in [55].

Young’s modulus of quartz has a sharp minimum in its temperature relationship at 573 °C [18,19]. The other components of the mixture (glassy phase and feldspar) have smooth dependencies of Young’s modulus on the temperature. Very similar behavior was also found for porcelain tiles [56] and ceramics with a high content of cristobalite [57]. Oliveira et al. [56] presented that the V-shape minimum of Young’s modulus during the cooling of ceramic tiles was attributed to quartz properties. The V-shape minimum was explained with the help of the mixture rule. This rule was used to calculate Young’s modulus of the theoretical curve for ceramic tiles in which Young’s modulus of quartz and glassy phase was used.

When the β → α transition of quartz is over, cracking continues down to the room temperature because the differences between LTEC of the sample components remained significant. This was reflected in the decrease of Young’s modulus (Figure 4) as well as in the presence of acoustic emission signals observed in previous studies [6,8,9,13,58].

The final values of Young’s modulus, the bulk densities, and the differences between the final and initial bulk density Δ*ρ* = *ρ_final_* – *ρ_initial_* are given in Table 4.

Table 4 shows that Kunda clay had the highest Young’s modulus and bulk density, which was reached by the most intensive sintering, as the highest value of Δ*ρ* suggests. The value of the Young’s modulus of the Kunda clay did not decrease during cooling after the β → α transition of quartz. Radobica and Liepa clays were sintered less, so their porosity did not change significantly. Their Young’s moduli after firing were only 3 times higher than Young’s moduli of the green samples. The final Young’s moduli of the investigated clays were scattered. Young’s modulus is hardly predictable from the bulk density—the value order of the bulk density differs from the order of Young’s modulus values. Young’s modulus results indirectly indicated that the original properties of the raw clay, mainly mineral composition and grain size distribution, play an important role in the mechanical properties of the final ceramic body.

**Figure 7:**
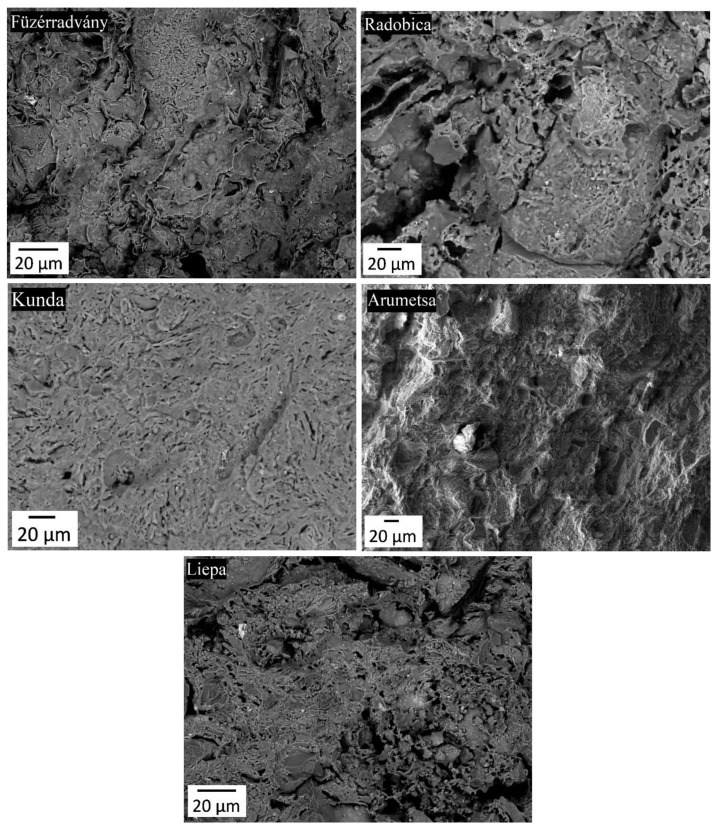
The SEM micrographs of fracture surfaces of experimental samples fired at 1100 °C.

SEM pictures of the fracture surfaces taken after firing at 1100 °C are shown in Figure 7. A relatively high porosity is visible in the pictures, except for the Kunda clay, which has a fine microstructure and no large defects. This partially explains high Young’s modulus of the Kunda clay, which keeps its value during cooling down to the room temperature (Figure 4). On the other hand, the Radobica and Liepa clays are characterized by significant porosity and cracks around the quartz grains, leading to low Young’s modulus.

## 4. Conclusions

Results of D-TMA for five illite-based clays from deposits in Slovakia, Estonia, Latvia, and Hungary were presented. The clays consist of illite (37–80 mass%), quartz (12–48 mass%), K-feldspar (4–13 mass%), kaolinite (0–18 mass%), and calcite (0–3 mass%). The shape and the dimensions of the samples were very close to each other, therefore significant differences between the results due to the sample volume and shape was not considered. The analyses focused on Young’s modulus measured during the cycle 20 °C → 1100 °C → 20 °C. It was found that:

- The release of the physically bound water increases Young’s modulus by ∼70%.
- The influence of the α → β quartz transition and dehydroxylation of illite on Young’s modulus is negligible during heating.
- The intensive sintering, which takes place at ∼800 °C → 1100 °C → 800 °C increases Young’s modulus.
- Solidification of the glassy phase is finished at ∼750 °C. Cooling from this temperature, the creation of cracks begins due to differences between the thermal expansions of quartz, glassy phase, and other mineral phases.
- At around the β → α quartz transition, a partial recovery of Young’s modulus occurs as the result of the thermal stresses reversal.
- Young’s modulus lowers its values down to the room temperature as the consequence of cracking.
- The results of Young’s modulus indicate that the mineral composition and character of the clay particles, determined by the clay’s origin, play an important role for Young’s modulus, with the final values varying between 15 GPa to 68 GPa.
- Only the Kunda clay from Estonia keeps its Young’s modulus values after the β → α quartz transition. To explain this anomalous behavior, a new set of experiments should focus on studying the microstructure, composition, and granulometry of the Kunda clay.

## Figures and Tables

**Table 1:** Mineral composition of the investigated clays (in mass%) [7,10,11,12,13].

Minerals	Füzérradvány	Radobica	Kunda	Arumetsa	Liepa
Illite	80	51	54	43	37
Kaolinite	–	–	8	18	15
Montmorillonite	4	–	–	–	–
Chlorite	–	–	5	–	–
Quartz	12	34	28	25	35
Feldspar	4	13	5	11	13
Calcite	–	2	–	3	–

**Table 2:** Chemical composition of the investigated clays (in mass%) [7,10,11,12,13].

Oxides	Füzérradvány	Radobica	Kunda	Arumetsa	Liepa
SiO_2_	58.4	56.7	61.4	57.8	62.7
Al_2_O_3_	23.9	23.1	17.8	18.7	15.9
Fe_2_O_3_	0.6	6.3	5.7	7.0	7.2
TiO_2_	–	0.5	–	–	1.9
CaO	0.4	0.4	0.4	1.6	0.9
MgO	1.7	2.4	2.3	2.6	1.5
K_2_O	7.7	5.0	5.6	4.8	4.3
Na_2_O	0.1	–	0.1	0.6	0.1
SO_2_	–	–	1.7	–	–
L.O.I	7.2	5.6	5.0	6.9	5.5

**Table 3:** Mineral composition of the investigated clays after firing at 1100 °C (in mass%) [7,10,11,12,13].

Minerals	Füzérradvány	Radobica	Kunda	Arumetsa	Liepa
Quartz	11	34	23	24.3	35
Feldspar	6	10	7	4.6	5
Hematite	–	4	2	3.0	4
Spinel	4	7	–	2.8	–
Amorphous	79	45	68	62.0	56

**Table 4:** The final values of Young’s modulus, the bulk densities, and the difference between the final and initial bulk density.

Quantity	Füzérradvány	Radobica	Kunda	Arumetsa	Liepa
*E_final_* (GPa)	39	17	68	30	15
*ρ_final_* (g/cm^3^)	1.84	1.95	2.27	2.17	1.94
Δ*ρ* (g/cm^3^)	0.18	0.1	0.44	0.28	0.08
